# Variable density dependence and the restructuring of coral-reef fisheries across 25 years of exploitation

**DOI:** 10.1038/s41598-018-23971-6

**Published:** 2018-04-10

**Authors:** Peter Houk, Javier Cuetos-Bueno, Brent Tibbatts, Jay Gutierrez

**Affiliations:** 1 University of Guam Marine Laboratory, UOG Station, Mangilao, GU 96923 Guam; 2 Guam Department of Agriculture, Division of Aquatic and Wildlife Resources, Mangilao, GU 96913 Guam

## Abstract

Variable density dependence within multispecies fisheries results in species restructuring as exploitation intensifies that is poorly understood. We examined unique species-based records across 25 years of exploitation to evaluate patterns, consequences, and predictions of species replacements within three coral-reef fisheries. Body-size was an expected determinant of species replacements, as larger fishes were consistently replaced by smaller, faster-growing counterparts. However, many species with similar sizes and growth rates responded differently. *Naso unicornis*, a primary component of coral-reef fisheries across the Pacific, was one of the most resilient species to exploitation despite having a similar maximum size and growth as many large parrotfishes that slowly disappeared from landings. Assessments conducted for all primary target species revealed clear distinctions in compensatory responses: 31% had diminishing size structures, 18% had diminishing proportional contribution, but only 5% showed both. Standard approaches to fisheries management assume constant rates of size-and-age restructuring and rely upon metrics such as fishing-versus-natural mortality. Instead, a deeper appreciation for varying recruitment rates may help to (re)define fisheries management units and reduce complexity in multispecies fisheries. We last consider our results alongside traditional knowledge and management in the Pacific that clearly appreciated species responses, but have been lost over the years.

## Introduction

Commercial coral-reef fisheries comprise hundreds of species, but a small subset contribute disproportionally to landings even on diverse Pacific reefs^1–3^. This common feature of reef fisheries provides an opportunity to simplify management within a complex system. Yet, developing formal stock assessments for even 20 species is well beyond the scope and financial means of most island nations that rely upon reef fisheries for their livelihoods. In response, a growing reliance is placed on indicators of fisheries status. Most fisheries-dependent indicators are based upon (extensive) snapshots at one point in time, comparing metrics such as size-at-capture versus optimal catch size and reproductive size, or use some combination of size-and-age-based criteria that estimate fishing mortality or reproductive potential^1,4–6^. The general premise is that target species decrease in size and age with fishing pressure, and quantifying where any population resides along a natural-versus-fishing-mortality gradient can provide an ideal metric of status. However, recruitment and growth differ widely across target species^7,8^. While density-dependent responses are fundamental principles of population ecology, the variation in their magnitude may select for species that can become dominant through time as fishing pressure increases, and conversely, select against those that diminish or even disappear from landings^9^. This situation causes shifting baselines, or reference points, that are not new to fisheries or coral reefs^10,11^; nor are the difficulties of predicting the outcomes of complex trophic interactions when species replacements occur^12^. But without historical data the fisheries-management framework often fails to address these issues, instead focusing on species with higher yields that have become dominant components of landings. This is a concern for coral reefs because ecological functions, economic values, and social benefits suffer as larger species with slow growth rates disappear and biomass turnover rates increase within the system.

Time-series data for coral-reef fisheries are rare and often disparate. Long-term investigations have dealt mainly with total landings and catch-per-unit-effort (CPUE), comparing and reconstructing trends across decades^3,13–15^. These studies have portrayed declines in landings across many Pacific Islands despite technological advances and expansion to new fishing grounds. Technological advances represent fixed-income assets that serve as a ratchet to commercial fisheries because growing fishing footprints are required to meet financial obligations^16,17^. Expansions can also mask localized depletions because supply keeps up with demand while footprints expand, painting a picture of sustainable stocks when examining commercial records of total landings. Shifts in target species eventually occur because fisheries are limited by habitat availability. However, our appreciation for species replacements in coral-reef fisheries remains limited by logistical difficulties and costs associated with accumulating species-specific data through time. The few temporal analyses at the family-or-trophic level have reported declines in accordance with trophic position (i.e., fishing down the food web), and identified disproportional targeting of some desirable herbivores families such as rabbitfishes and parrotfishes^18,19^. Meanwhile species-specific responses are depicted in fewer novel datasets derived from sources such as restaurant menus, photographs of historical fishing competitions, and rare governmental statistics reports^11,20,21^. This is troubling because less appreciation exists for the gradual replacement of species as baselines slowly shift through time^22^, and consistent supply to commercial markets masks localized depletions.

Temporal, fisheries-independent studies are more numerous and describe how fish assemblages track environmental cycles and respond to exploitation and management^23–26^. However, sample sizes for target species near human populations are usually low in visual assessments compared to catch landings, leading to management benchmarks that compare observed biomass to expected, or ‘unfished’, biomass^27–29^. These beneficial benchmarks can readily be monitored through time to assess the status of fisheries, but they do not necessarily link with species-specific management criteria for policy development. Coupling these benchmarks with a deeper appreciation for how species respond to fishing pressure may help ecosystem-based management evolve, and tailor policies to biological traits to maximize their effectiveness.

Here, we use a unique fisheries-dependent dataset from Guam, Micronesia, to examine responses to exploitation over a 25-year period. While the data are specific to Guam, the patterns of exploitation are generalized across three major fishery sectors to reveal common responses and hypothesize a common evolution of coral-reef fisheries subjected to commercial demand. Investigations at the family level were first conducted to determine what trophic groups may be most sensitive to fisheries expansions, what groups show a compensatory response, and to draw relationships with previous findings. We then focus within each major family to examine species replacements through time. Species replacements were examined with respect to size structures and proportional contributions to landings, representing two distinct features of both fisheries-dependent data and commercial sales. We last build a framework to evaluate target species that comprised 70% of the landings and discuss species responses with respect to modern and traditional forms of management.

## Methods

**Figure 1:**
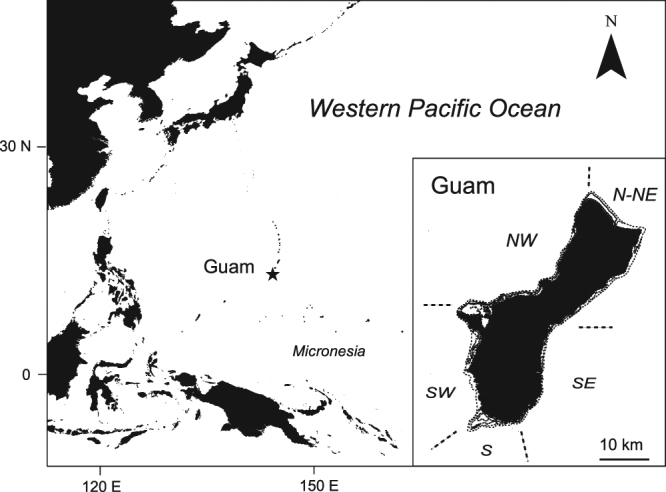
Map of the Western Pacific Ocean and Guam. Inset map shows the geographical quadrants used by the creel data collection program during the study period. Map was created by author PH using the ArcGIS v.10.2.2 software (http://www.esri.com/arcgis/about-arcgis).

Guam is the population center of Micronesia, located in the tropical North Pacific Ocean. Like many Pacific island societies, Guam has a cultural history tied with fisheries resources. However, colonization of Guam during the historical Spanish, German, and Japanese periods, and their current affiliation with the United States have decreased traditional management of their reef systems. Currently, Guam has the highest human population per-reef-area in Micronesia (1525 people km^−2^ for shallow reef habitat to 20 m depth)^30^, with a limited lagoon system and mainly fringing reefs surrounding the island (Fig. 1). The growing human population and tourism industry together place a strain on limited coastal fisheries resources^31^. Recent fisheries-independent studies have suggested Guam had the second lowest fish biomass among 23 island nations, and up to 3 times lower than remote islands in the same island chain^32^. Recent fisheries-dependent studies on Guam’s coastal fisheries have: (i) characterized the local fishery sectors, (ii) described how consistent effort but declining landings have evolved between 1985 and 2012, and (iii) described a general shift in assemblages from large parrotfishes to mixed acanthurids in Guam’s SCUBA fishery thorough time, and (iv) found reduced sizes of most common target species compared to other Micronesia islands^1,3,19,33^. These studies set the stage for the present analysis that examined species sizes, proportional contributions to landings, and species replacements across three fisheries sectors exposed to growing exploitation.

### Data collection

**Figure 2:**
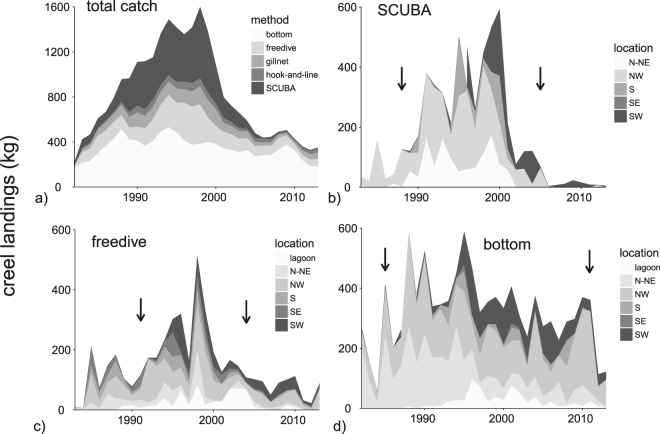
Reported landings from the creel data collection program broken down by method (**a**) and location (**b**–**d**) for the top three fisheries examined. Locations refer to geographical island sectors (Fig. 1). Arrows indicate the timeframes considered in the analysis of each fishery, described in the methods by a 3-year rolling average of 50 kg yr^−1^ or more.

The datasets analyzed during this study are available upon request to the Guam Division of Aquatic and Wildlife Resources (DAWR). Guam DAWR established a fisheries-dependent monitoring program in 1982 that targeted both commercial and recreational fishers. DAWR staff intercepted fishers as they return from both boat-based and shore-based trips. Creel surveys followed a regular schedule, including shifts on weekdays, weekends, and evenings when peak fishing periods were noted for each of the fishery sectors. During each survey event, all fish were identified and measured to the nearest mm fork length, and a series of standard questions were asked to determine fishing location(s) and method(s). Species-based data were then entered into a standardized database that is available upon request to the Guam DAWR. While several sectors were covered by this program, SCUBA spearfishing (to ~90 m), freedive spearfishing (to ~18 m), and shallow bottom fishing (to ~90 m) were most common and selected for analyses based upon data availability (Fig. 2). The stream of data from this program differs for each fishery, but reporting was highest and most consistent between the late-1980’s and mid-2000’s. Reporting for the SCUBA and freedive fishery greatly diminished in the mid-2000’s due to controversy among fishers, managers, and stakeholders. Reporting for the bottom fishery diminished in 2010 as the local program reduced their staff and coverage, and a complimentary federal program initiated. We used a minimum rolling average of 50 kg yr^−1^ to determine the timeframe for analyzing each fishery (Fig. 2b–d), and applied weighted regression models to take varying reported landings into account (*see data analyses*). This provided a conservative approach to account for increasing variances associated with decreasing sample sizes.

The United States National Marine Fisheries Service Pacific Islands Program began collecting similar species-based catch records and complimentary life-history data in 2010 across all US affiliated territories in the Pacific, including Guam. While comparable daily landing data are available from this program to potentially extend our time-series analyses, these data have been determined to represent confidential data that are not accessible to the public. The request for the data and for confidential aspects of the data to be removed was not approved (request by author PH).

### Data processing

The DAWR dataset was first filtered to include records from the SCUBA, freedive, and bottom fishing sectors (Fig. 1). We then applied a series of additional filters to extract the main components of the database by removing: (i) families that contributed <1% to overall landings (2% of data), (ii) species that contributed to <1% of family landings (2% of data), and (iii) fishing locations outside of Guam. We last removed any records that included obvious errors in fish measurements or fishing methods (<1% of data). This process resulted in a final database including 131 species, 78,347 fishes, and 30,730 kg.

Species attributes were next integrated into the database. We used a hierarchical process to determine length-to-weight coefficients for all species, using data derived from: 1) Guam (49%^34,35^;), 2) Commonwealth of the Northern Mariana Islands (nearest geographic neighbor with similar island geology; 9%^36,37^;), 3) ongoing fisheries-dependent research elsewhere in Micronesia (4%; unpublished data author JCB), and 4) other regions in the Pacific (38%^38^;). We also classified fishes within main target families into body-size categories based upon their maximum lengths using Jenks breaks (Supporting Information 1). This process grouped species into 2 or 3 size categories, depending upon the range of sizes in the family. Large-to-small fish ratios were then calculated by dividing the biomass of fish from the larger categories divided by the smallest.

### Data analyses

Time series analyses were conducted across the study period using moving averages of the dependent variables^39^. We assumed years across the study period represented an exploitation gradient given the steady decline in catch success despite consistent effort reported for Guam across our study period^3^, and few changes to fisheries management policies that were centered on creating no-take marine protected areas in 1997 in response to declining fish stocks^40^. Moving averages were calculated based upon three sequential timesteps, before-during-after each study year. This window size was selected to minimize autocorrelation of residuals and maximize the length of data records used for regressions. Because moving averages of dependent variables were calculated, the resultant model parameters were only used for comparative purposes and not to evaluate the absolute magnitude of trends. Examples include contrasting size changes versus proportional contribution changes through time (i.e., did a species decline more in size or proportional contribution to landings over the years?). Weighted regression models were used to assess trends. Weighted models attribute added values, or weights, to each data point based upon confidence of the information^41^. Here, confidence was defined by the total weight of fish or numeric density, depending upon whether biomass or abundance data were used. Three models were examined for each regression analysis representing realistic fishery scenarios. First, linear models inferred sequential decreases or increases in biomass, density, or size over the study period. Second, exponential models inferred strong non-linear increases or decreases, followed by weaker saturating responses, often reported for sensitive fishery species that respond quickly to exploitation or compensation. Third, polynomial models inferred humped relationships, whereby a maximum or minimum may be reached, followed by the opposite trend. These may be relevant for expanding fisheries, with humps in catch/size being related to fisheries expansions or retractions. Best fit models were considered based upon AIC values that essentially balanced model fit against residual normality (i.e., R^2^ values and their associated P-values versus the results from a Shapiro-Wilks normality test of residuals). Residuals were also examined for autocorrelation to ensure the variances were constant through time^42^. Analyses were conducted using R^43^.

One notable fishery expansion occurred during the study period when a new boat marina on the southwestern side of Guam was constructed in 1995, providing novel access to the south and southwest geographic sectors. The influence of this expansion on temporal trends was assessed through preliminary analyses of mean fish sizes with respect to geographic sectors (Supporting Information 2). Increased sizes were noted for both the bottom and freedive fishery following the fishery expansion. Given significant findings, arrows were placed on each figure to indicate the 1995 expansion, and appreciate this unique event in the time series analyses.

For all three fisheries, we analyzed several dependent variables sequentially: 1) percent contribution of dominant fish families (>10% of landings), 2) ratios of large-to-small bodied fishes within each dominant family, 3) mean size for all ‘other fishes’ not in dominant families, and 4) percent contribution of large, iconic species grouped together. Large, iconic species included the Napoleon wrasse (*Cheilinus undulates*), bumphead parrotfish (*Bolbometopon muricatum*), giant trevally (*Caranx ignobilis*), dogtooth tuna (*Gymnosarda unicolor*), and giant grouper (*Epinephelus lanceolatus*). We then contrasted SCUBA versus freediving sectors because they occur in similar habitats, target similar species, and may have linked trends. Target species were binned into functional groups (i.e., large-bodied parrotfishes), and standard pairwise comparisons were made.

Last, to better appreciate species-based responses and potential replacements, time series analyses were performed on mean size and proportional contribution for dominant species that made up 70% of landings from their respective fishery. The results were then used to assign ‘potential management’ categories. Size-based policies were considered best options for species with stronger impacts to their size structure compared to their proportional contributions. Gear/quota/area policies were considered best for species that slowly fade out of the fishery with less impact to their size structure^44^. Species were assessed using the following criteria: 1) asymptotic or polynomial models with immediate declines were considered stronger than linear models, 2) if similar regression models existed between size and contribution trends, differences in effect sizes with non-overlapping standard errors were used, 3) if similar models existed but effect sizes were overlapping, then both potential management strategies were considered equal, and 4) species with no significant responses or increases through time were not placed into any potential management category.

## Results

### Bottom fishery

**Figure 3:**
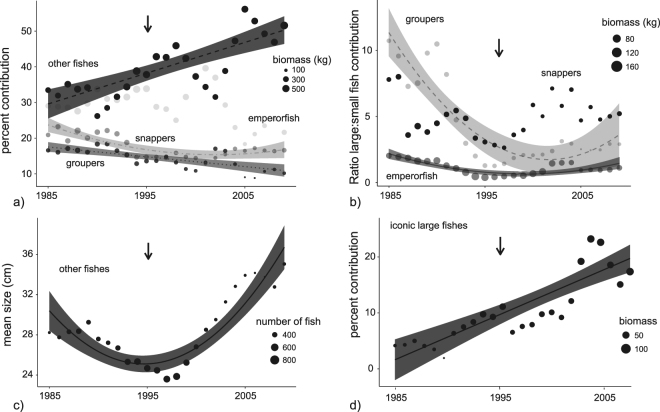
Temporal trends in the shallow bottom fishery. Trends in proportional contributions and large-to-small fish ratios are shown for dominant families (**a**,**b**). Remaining target species not included in family-level analyses were grouped into ‘other fishes’ or ‘iconic large fishes’ (**c**,**d**). Data points represent mean values for each year based upon a moving three-year smoother. Data points were scaled by sample sizes that were used in weighted regression models (i.e., circle sizes). Confidence bands are only shown for significant relationships (Table 1). Arrows indicate the expansion of the fishery when a new boat marina was constructed in 1995.

The bottom fishery was the largest and most consistent contributor to overall landing reported in the governmental creel program (Fig. 2a,d). Bottom fishing focused mainly on snappers (14 to 25% contribution across the study years), groupers (8 to 18%), emperorfishes (30 to 40%), with smaller contributions from trevallys, goatfishes, squirrelfishes and soldierfishes (noted as ‘other fishes’ within analyses, Fig. 3). Significant declines in the proportional contributions of both snappers and groupers were found across the 25-year period (30% and 45% net decline, respectively, Fig. 3a, Table 1). In contrast, emperorfish contributions fluctuated nonuniformly, while ‘other fishes’ increased linearly by 75%. The bottom fishery responded positively to the fishery expansion in 1995, as mean fish sizes in the newly accessible areas were larger compared with others for several years following the construction of a new boat marina (Supporting Information 2). Thus, the expansion was associated with polynomial best-fit regressions for the size-based dependent variables examined (Table 1). The ratio of large-to-small groupers declined strongly during the first ten years, but showed a slight positive response to the fishery expansion in 1995 (90% net decline, polynomial regression, Fig. 3b, Table 1). These trends were driven by reduced contributions from two large species, *Variola louti* and *Cephalopholis sonnerati*, and replacement by *Epinephelus fasciatus* and other small-bodied groupers (Table 2). Similarly, the ratio of large-to-small emperorfishes decreased steadily until 1995 mainly driven by a decline in *Lethrinus rubrioperculatus* (50% decline). The ratio increased slightly following the expansion with higher proportional contributions from *L. xanthochilus*. Meanwhile, the ratio of large-to-small snappers did not differ across the study period, however species replacements were noted. Proportional contributions from *Aprion virescens* became rare while *Lutjanus bohar* became more common after the expansion, despite the latter being a ciguatoxic species that causes human sickness (i.e., potential bycatch, Fig. 3b, Table 2). Mean size within the collective group of ‘other fishes’ also declined between 1985 to 1995, but showed a significant increase with growing contributions from reef-pelagics such as barracuda (*Sphyraena* spp., Fig. 3c, Table 2). Last, contributions from large iconic species increased linearly across the entire 25-year period as increases in a large reef-pelagic species, the dogtooth tuna, were evident (*Gymnosarda unicolor*, Fig. 3d, Table 2).

**Table 1:** Best-fit regression models that described time series trends for the three dominant fisheries sectors depicted in Figures 3 to 5. Methods describe the model selection process. P-values < 0.05 (*), <0.01 (**), <0.001 (***).

Fishery	Fish group	Dependent variable	Equation	R2 and P-value	Figure
Bottom	snapper	% contribution	y = −1.1x + 0.03x^2^ + 24.7	0.64^***^	2a
Bottom	grouper	% contribution	y = −0.27x + 17.8	0.51^***^	2a
Bottom	other fish	% contribution	y = 0.9x + 28.7	0.62^***^	2a
Bottom	grouper	large:small ratio	y = −0.17x + 0.005x^2^ + 2.2	0.57^***^	2b
Bottom	emperorfish	large:small ratio	y = −0.91x + 0.02 x^2^ + 11.9	0.74^***^	2b
Bottom	other fish	mean size	y = −1.2x + 0.06x^2^ + 31.6	0.78^***^	2c
Bottom	large iconic fish	% contribution	y = 0.8x + 0.2	0.67^***^	2d
SCUBA	parrotfish	% contribution	y = −2.6x + 0.09x^2^ + 49.7	0.5^**^	3a
SCUBA	other fish	% contribution	y = 2.1x − 0.07x^2^ + 13.2	0.77^***^	3a
SCUBA	parrotfish	large:small ratio	log(y) = −0.6 log(x) + 2.5	0.49^**^	3b
SCUBA	surgeonfish	large:small ratio	y = −0.6x + 0.05x^2^ + 3.7	0.87***	3b
SCUBA	grouper	large:small ratio	log(y) = −2.8x + 43.1	0.45^**^	3b
SCUBA	others	mean size	log(y) = −0.05 log(x) + 3.4	0.64^***^	3c
SCUBA	large iconic fish	% contribution	y = −4.9x + 0.3x^2^ + 35.3	0.75^***^	3d
Freedive	parrotfish	% contribution	log(y) = −0.2 log(x) + 3.7	0.79^***^	4a
Freedive	surgeonfish	% contribution	y = 0.9x + 23.9	0.64^**^	4a
Freedive	other fish	% contribution	y = 2.6x − 0.2x^2^ + 33.6	0.41^*^	4a
Freedive	surgeonfish	large:small ratio	y = 0.11x + 0.74	0.82***	4b
Freedive	other fish	mean size	y = −0.5x + 0.02x^2^ + 23.9	0.53^**^	4c

**Table 2:** Trends in size and proportional contribution for target species that comprised the majority of bottom fish landings, with sample sizes ≥50. Methods describe potential management criteria.

Species	N	Proportional biomass	Size	R2 (P-value)	Percent contribution	R2 (P-value)	Potential management
Lethrinus rubrioperculatus	2977	11.4		0.16*		0.83^***^	gear/quota/area
Aprion virescens	397	8.8		0.46***		0.70^***^	gear/quota/area
Gymnosarda unicolor	165	6.5	—	—		0.56^***^	—
Epinephelus fasciatus	2338	5.4		0.80**		0.69^***^	size
Lethrinus xanthochilus	605	4.4	—	—		0.74^***^	—
Carangoides orthogrammus	340	3.6	—	—	—	—	—
Lethrinus olivaceus	286	3.4	—	—	—	—	—
Caranx lugubris	150	3.3	—	—	—	—	—
Variola louti	536	3.2	—	—		0.21^*^	gear/quota/area
Seriola dumerili	77	3.1		0.45**		0.54^***^	size
Lethrinus obsoletus	1098	3.1		0.24*		0.50^***^	size
Caranx ignobilis	75	2.6		0.58***		0.56^***^	—
Caranx melampygus	191	2.6		0.26**		0.57^***^	size
Lutjanus bohar	119	2.6		0.15*		0.41^**^	—
Sphyraena genie	71	2.3	—	—		0.70^***^	—
Cephalopholis sonnerati	324	2.3		0.50***		0.65^***^	both/either
Lethrinus harak	831	2.2		0.15*		0.46^***^	size

### SCUBA and freedive fisheries

**Figure 4:**
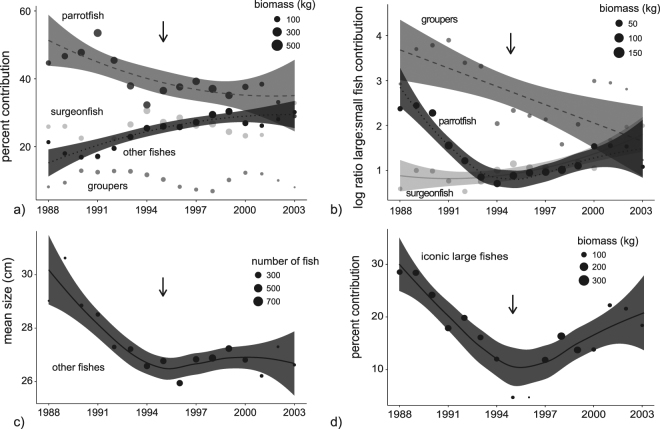
Temporal trends in the SCUBA fishery. Trends in proportional contributions and large-to-small fish ratios are shown for dominant families (**a**,**b**). Remaining target species not included in family-level analyses were grouped into ‘other fishes’ or ‘iconic large fishes’ (**c**,**d**). Data points represent mean values for each year based upon a moving three-year smoother. Data points were scaled by sample sizes that were used in weighted regression models (i.e., circle sizes). Confidence bands are only shown for significant relationships (Table 1). Arrows indicate the expansion of the fishery when a new boat marina was constructed in 1995.

The SCUBA fishery was the second largest contributor to overall landing with a peak in landings reported between the late 1980’s and mid 2000’s (Fig. 2a,b). SCUBA fishing focused mainly on parrotfishes (35 to 50% contribution across study years), surgeonfishes (15 to 28%), and groupers (4 to 14%), with smaller contributions from numerous other families (i.e., ‘other fishes’). The expansion in 1995 had no influence on mean fish sizes which declined uniformly across geographic sectors (Supporting Information 2). Parrotfish had the most notable decline in proportional contribution (30% net decline), while surgeonfish and groupers fluctuated non-significantly (Fig. 4a). In response, the collective group of ‘other fishes’ steadily increased their contribution to landings. Although ‘other fishes’ steadily grew in contribution, they experienced an asymptotic decline in size that flattened out shortly before the fisheries expansion (Fig. 4c). The ratio of large-to-small groupers and parrotfishes both declined strongly across the entire study period (over 20-fold decline for both, log-scale used, Fig. 4b, Table 3). Large parrotfish declines were driven by reduced *Hipposcarus longiceps*, *Scarus altipinnis*, *Chlorurus microrhinos*, and *Scarus rubroviolaceus* landings (Table 3). In response, small parrotfishes that responded positively through time were *Chlorurus spilurus*, *Scarus schlegeli*, and a mixture of other species. Groupers contributed less to the SCUBA fishery compared to the bottom fishery, but declining large-to-small ratios were similarly driven by reduced contributions from *Varoli louti* and *V. albimarginata* to a lesser extent, which were replaced by the smaller *Epinephelus fasciatus* (Table 3). Uniquely, the ratio of large-to-small surgeonfishes increased across the entire study period with growing contributions from the iconic *Naso unicornis*. Last, iconic large fish contributions declined asymptotically by 60%, with a slight increase following the fisheries expansion (Fig. 4d). Iconic large fishes in the SCUBA fishery were disproportionally represented by the Napoleon wrasse, *Cheilinus undulatus*.

**Table 3:** Trends in size and proportional contribution for target species that comprised the majority of SCUBA landings, with sample sizes ≥50. Methods describe potential management criteria.

Species	N	Proportional biomass	Size	R2 (P-value)	Percent contribution	R2 (P-value)	Potential management
Naso unicornis	700	11.2		0.39**	—	—	—
Hipposcarus longiceps	381	9.3		0.84***		0.23^*^	size
Scarus altipinnis	343	6.5		0.73***		0.28^*^	gear/quota/area
Chlorurus microrhinos	255	4.8		0.45**		0.82^***^	gear/quota/area
Scarus schlegeli	416	3.3		0.64***		0.69^***^	size
Scarus rubroviolaceus	111	2.8		0.85***		0.82^***^	gear/quota/area
Naso lituratus	589	2.6		0.70***		0.60^***^	both/either
Monotaxis grandoculus	137	2.2	—	—		0.31^*^	—
Chlorurus spilurus	369	2.1	—	—		0.27^*^	—
Naso caesius	94	1.6	—	—	—	—	—
Epinephelus polyphekadion	50	1.6	—	—	—	—	—
Variola louti	67	1.4		0.54***	—	—	size
Caranx melampygus	51	1.3		0.60***		0.52^**^	size
Siganus punctatus	197	1.3		0.52***		0.32^*^	size
Scarus forsteni	115	1.3	—	—		0.38^*^	gear/quota/area
Acanthurus xanthopterus	124	1.2	—	—	—	—	—
Siganus argenteus	284	1.1		0.64***		0.76^***^	both/either

**Figure 5:**
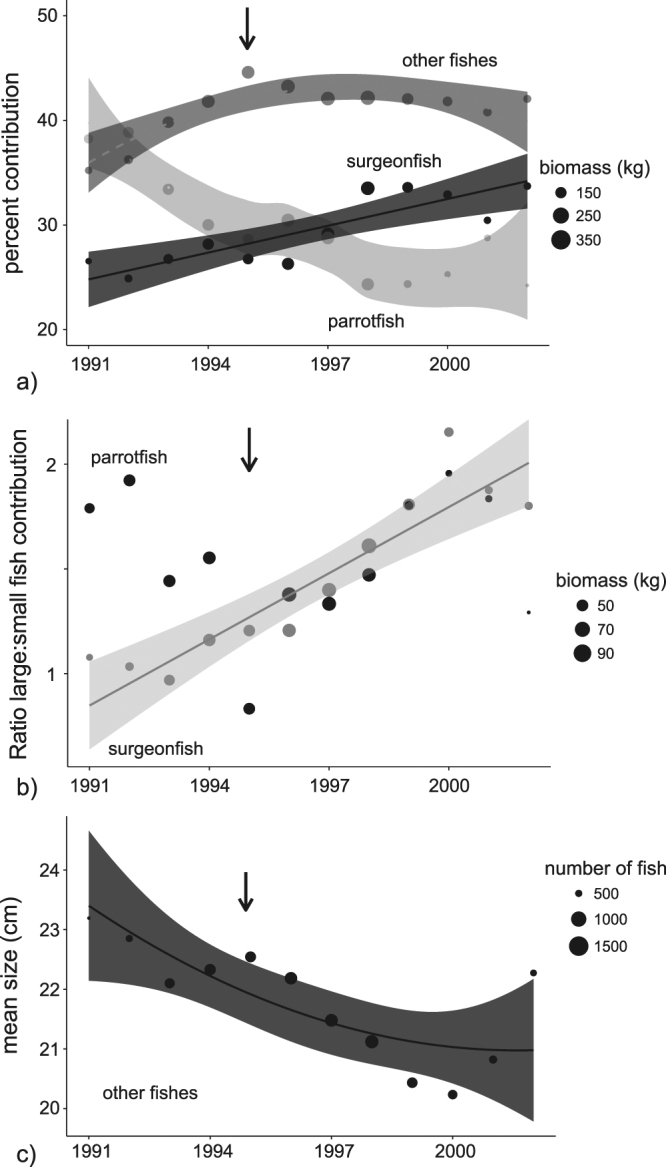
Temporal trends in the freedive fishery. Trends in proportional contributions and large-to-small fish ratios are shown for dominant families (**a**,**b**). Remaining target species not included in family-level analyses were grouped into ‘other fishes’ (**c**). Data points represent mean values for each year based upon a moving three-year smoother. Data points were scaled by sample sizes that were used in weighted regression models (i.e., circle sizes). Confidence bands are only shown for significant relationships (Table 1). Arrows indicate the expansion of the fishery when a new boat marina was constructed in 1995. Large iconic species were rare in the freedive fishery and no trends were detected (graph not shown).

The freedive fishery was the third largest contributor to landing with peaks reported between the early 1990’s and mid 2000’s (Fig. 2a,c). Freedive fishing focused mainly on parrotfishes (25 to 40% contribution across study years) and surgeonfishes (25 to 35%), with smaller contributions from numerous other families. There was a significant decline in parrotfish contributions which were replaced by both surgeonfishes and ‘other fishes’ (Fig. 5a, Table 1). Large-to-small parrotfish ratios did not change across the years, yet species shifts were detected. Declines in contribution from *Chlorurus microrhinos* and *Scarus rubroviolaceus* were accompanied by non-significant increases for *Chlorurus frontalis* and *Hipposcarus longiceps* (Table 4). In contrast, large-to-small surgeonfish ratios increased slightly from 1 to 2 with growing contributions from *Naso unicornis* and *Acanthurus xanthopterus*. Yet, ratios of large-to-small surgeonfishes and parrotfishes were both depressed below 2 during the entire study period, highlighting strong differences between the freedive and SCUBA fishery that targeted similar species (Fig. 5b compared with Fig. 4b, note the log scale in the latter). There was a steady decline in the size of ‘other fishes’ in the freedive fishery that was less indicative of species replacements and better attributed to declining sizes of numerous target species (Fig. 5c). Last, large iconic species were rare and no trends were detected.

**Table 4:** Trends in size and proportional contribution for target species that comprised the majority of freedive landings, with sample sizes ≥50. Methods describe potential management criteria.

Species	n	Proportional biomass	Size	R2 (P-value)	Percent contribution	R2 (P-value)	Potential management
Naso unicornis	679	12		0.82***		0.67^**^	size
Naso lituratus	886	5.8		0.81***	—	—	size
Chlorurus frontalis	215	4.5	—	—	—	—	—
Chlorurus spilurus	473	4.3	—	—	—	—	—
Chlorurus microrhinos	136	4.1		0.67***		0.61^***^	gear/quota/area
Scarus schlegeli	368	3.8		0.50**		0.59^**^	size
Kyphosus cinerascens	147	3.7		0.73***	—	—	—
Acanthurus lineatus	522	3.4		0.74***	—	—	size
Hipposcarus longiceps	167	3	—	—	—	—	—
Scarus psittacus	323	2.8		0.81***		0.23^*^	size
Kyphosus vaigiensis	99	2.6		0.40*	—	—	—
Cheilinus trilobatus	243	2.6		0.25*		0.35^*^	size
Scarus rubroviolaceus	67	2.5		0.61**		0.41^*^	gear/quota/area
Siganus spinus	526	2	—	—		0.93^***^	—
Acanthurus xanthopterus	160	2	—	—		0.97^***^	—
Epinephelus merra	383	1.9		0.64**	—	—	—
Parupeneus barberinus	243	1.7	—	—		0.60^**^	—
Caranx melampygus	57	1.7		0.64***	—	—	size
Scarus altipinnis	77	1.6	—	—	—	—	—
Acanthurus triostegus	424	1.1		0.61***		0.90^***^	—

**Figure 6:**
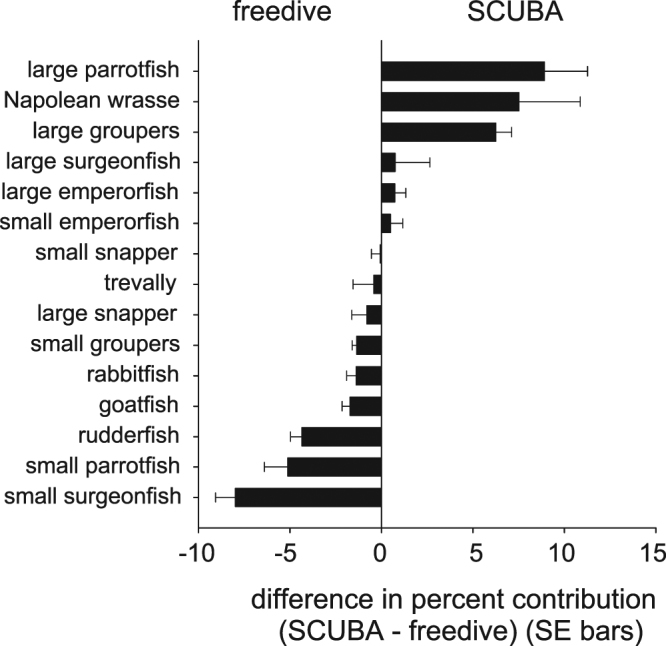
SCUBA versus freedive fishery comparisons. Differences in percent contribution for target fish categories. Data points represent mean values for each year based upon a moving three-year smoother. Data points were scaled by sample sizes that were used in weighted regression models (Table 1). Arrows indicate the expansion of both fisheries when a new boat marina was constructed in 1995, and when data from the SCUBA fishery on the N-NE sector of the island stopped being collected in 2002.

In sum, clear differences were evident in the composition of SCUBA versus freedive landings across the entire study period. SCUBA landings were composed of larger species including several parrotfishes, groupers, and the iconic Napoleon wrasse (Fig. 6). Freedive landings were represented by smaller species within these families, as well as rabbitfishes, goatfishes, and rudderfishes.

### Management responses

Fifty-four percent of target species were placed into management response categories (65%, 53%, and 45%, respectively for SCUBA, bottom, and freedive, Tables 2–4). Among species placed in management categories, 59% had strongest responses to their size structure, 31% had strongest responses to their proportional contribution, and 10% had equal responses. Species with significant responses to exploitation in more than one fishery had similar management categories in all but one instance (n = 6 species with significant responses in more than one fishery, only the grouper *Variola louti* differed between bottom and SCUBA (Tables 2–4).

## Discussion

Both common and unique species replacement trends were revealed within three coral-reef fisheries across two decades. We found that fishing ‘down’^45^ and ‘through’^46^ food webs occurred simultaneously, as larger fishes from differing trophic levels were primarily targeted. This suggested that fish size, rather than trophic position, was a primary driver of exploitation. Small differences in species-based sales prices across major target families support the simple desirability of catching larger fish to maximize economic benefits^47^. In turn, smaller species with faster growth rates responded over the years and grew in proportional contribution. Groupers and emperorfish were primary bottom-fish families with large-to-small species replacements, while removing the ciguatoxic *Lutjanus bohar* resulted in similar large-to-small species replacements for snappers as well. Groupers and parrotfish were primary SCUBA-fish families with large-to-small species replacements. Meanwhile, the contribution of parrotfish within the freedive fishery declined, but similar species replacements were not observed. This appeared to reflect different initial baselines, as large-to-small parrotfish ratios were already depressed below 2 at the start of the study, but were substantially higher for the SCUBA fishery in early years (i.e., log scale in Fig. 4b). However, when considering the changes in catch biomass by trophic level, fishing ‘down’ the food web also became apparent as herbivores and invertivores from a larger suite of families became more abundant. The net effect of selective fishing for larger species in higher trophic levels has been an increase in biomass turnover rates, and a previously reported decline in overall landings despite stable fishing effort (63% decline in landings estimated)^3^.

A second, common trend was the growing contribution from ‘other fishes’ (i.e., the suite of fish not in target families) that was mainly due to their persistence while target families declined and indiscriminant and opportunistic fishing pressure evolved. Interestingly, many fish had consistent, or even growing proportional contributions while their sizes diminished. Sustained or increased percent contributions despite reduced sizes depicted a classic compensatory density dependence response^48^, whereby removal of adults made space for greater abundances of juveniles (i.e., the rabbitfishes *Siganus spinus* and *S. punctatus*, the goatfish, *Parupeneus barberinus*, the small emperorfish *Lethrinus harak*, and several others, Tables 2–4). Yet, even species with strong density dependence can eventually decline in contribution given enough fishing pressure. Therefore, a deeper appreciation for variation in species-based responses added to the longstanding debate surrounding density dependence in fisheries^9^, and improved the way species could be grouped into management units based upon their expected responses to fishing pressure.

### Species-based responses

Generally, shifting size structures were reported for small species within their respective families, supporting the fundamental relationship between body size and growth rates^49^, and that faster growing species would be less susceptible to growing exploitation. However, several large herbivores also had primary responses to their size structure, such as the Pacific longnose parrotfish (*Hipposcarus longiceps*), the bluespine unicornfish (*Naso unicornis*), and rudderfishes (*Kyphosus* spp.), which were accompanied by both increasing and decreasing proportional contributions. These findings were unexpected given that growth rates of these species were similar to other large, late maturing parrotfishes that have been declining more in terms of contribution than size from many Pacific fisheries^1,8,44^. Considering that spawning potential is also well known to decrease with body size^5,6^, we hypothesized that size-based responses for these species were due to reduced post-settlement mortality and/or large population sizes rather than increased recruit production. In support, (i) both freedive and SCUBA fisheries had growing unicornfish contributions despite being the top landed species and having mixed size responses, (ii) unicornfish have been found to have high genetic connectivity, juvenile settlement, and post-settlement mortality^50–52^, and (iii) unicornfish represent primary target species across Pacific fisheries^1,44^. As life history knowledge for more target species evolves, it will clearly be interesting to simultaneously consider responses to fishing pressure to better inform fisheries projections and models. We hypothesize that life histories dictate how a species *can* respond to exploitation, but variation in recruit survival may represent an underappreciated, secondary driver of how a species *will* respond to exploitation. The present time series data were rare, but obviously useful for determining species patterns. Further development and examination of long-term landings data at higher taxonomic resolution than typically exists can test the generality of our findings. Data from other localities can also test whether species response patterns differ geographically, and potentially lead to generalized guidance for target species across the Pacific.

### Management

The results cautioned that unchecked exploitation could quickly remove the suite of ‘sensitive’ species that declined most in proportional contribution, and allow species with strong compensatory density dependence to replace them. The sensitivity of large and long-lived fishes to exploitation is well documented^11,53^, but we reported declining contributions from many mid-sized fishes that are currently well represented across Pacific fisheries (e.g., many parrotfishes). Compromised populations of these species have ecological and economic consequences, such as declining corals and calcifying substrates within reef ecosystems^54^, which can also impact the goods and services offered to society and tourism industries^55^. Therefore, it is desirable to know what species could be grouped into management units to simplify guidance for complex multispecies fisheries, and how the shared life histories and responses to exploitation within each group best link with management options. We reconciled that management units should be defined by trophic level, body-size (i.e., life history), and response categories, rather than taxonomy. For instance, several large parrotfishes with similar maximum body sizes all declined in proportional contribution across the study period suggesting quota/gear/area policies may work best (e.g., *Chlorurus microrhinos*, *Scarus altipinnis*, and *S. rubroviolaceus*), but should not be applied across all large parrotfishes because some showed strong density dependence and may better be managed by size limits (e.g., *Hipposcarus longiceps*). We tailor further management discussion with respect to Guam and the Pacific, to blend our results with both modern and traditional forms of management.

Large-to-small species replacements were consistent across most Guam fisheries. The only exception was the increase in bottom fish size following the fishery expansion, driven by a few reef-pelagic species such as barracuda and dogtooth tuna. The expansion may have provided greater access to migratory reef-pelagic species, or alternative fishing techniques within the bottom fishing category have become more available through time (i.e., jigging for larger reef-pelagic species). Generally however, the present findings agree with past studies describing the decline in Guam’s nearshore fishery through time, and also suggested disproportional impacts from the SCUBA fishery compared to others^3,19^. While limiting or restricting the use of SCUBA is one clear recommendation that resonates with past studies, holistic approaches towards management have been given less attention. Policies of the United States applied to Guam’s coral reefs during our study period, for example, have defined management units taxonomically by families^56^. Catch quotas rooted in the concept of maximum sustainable yields for families such as groupers or parrotfishes would have committed the system to species-based replacements we depicted. To improve upon this situation, we blended our results within a traditional management framework.

Traditional management started with resource ownership, or reef tenure that was common to Guam and the Pacific^57–60^, and established a series of spatially-disconnected fisheries units. However, management within each unit was similar, unless there were different habitats present. Limited entry policies defined fishing rights based upon social status and fishing methods. More than 50 different traditional fishing techniques were documented on Guam and the Mariana Islands to target certain species at specific timeframes, life stages, and sizes^61^. Similar policies also existed across the Pacific to limit fishing for large iconic fish, protect large spawning events, create no fishing taboo areas for reefs to recover, release certain species and sizes from fish traps, and shift fishing techniques with seasons^58,59,62^. The hierarchy and diversity of traditional management could only be derived through extensive species-specific knowledge which has been lost over the years. In replacement, modern fisheries management is heavily influenced by the concept of compensatory density dependence, estimations of natural-to-fishing mortality, and numerous derivatives of length-based metrics^5,63^. While length-based responses are obviously central to fisheries management, we add that a deeper appreciation for variable fish responses may improve holistic management approaches that resonate with traditional knowledge and are socially acceptable.

## Electronic supplementary material

Supporting Information
