# Supplementary material for: Variable density dependence and the restructuring of coral-reef fisheries across 25 years of exploitation
Source: Sci Rep. 2018 Apr 10;8:5725. doi: 10.1038/s41598-018-23971-6 (PMC5893587; doi:10.1038/s41598-018-23971-6)
Supplement: Supplementary file 1 — Supporting Information [file 41598_2018_23971_MOESM1_ESM.pdf]

Supplementary Information:

Variable density dependence and the restructuring of coral-reef fisheries across 25 years of exploitation

Authors: Peter Houk[*1], Javier Cuetos-Bueno[1], Brent Tibbatts[2], Jay Gutierrez[2],

Author Contributions: PH, JCB, BT, and JG designed study. PH and JCB performed analyses. BT and JG prepared the datasets. PH wrote the main manuscript text. PH, JCB, BT, and JG reviewed the manuscript.

Affiliations: [*1]University of Guam Marine Laboratory, UOG Station, Mangilao, GU. 96923; [2]Guam Department of Agriculture, Division of Aquatic and Wildlife Resources, Mangilao, GU. 96913

[*]**Corresponding author contact:** peterhouk@gmail.com; ph (671) 735-2188; fax (671) 734-6767

Family	*Species*	*body-size group*	*sample size (present study)*	L_{max} (cm)	*source*
Acanthuridae	*Acanthurus blochii*	small	146	34	1
Acanthuridae	*Acanthurus guttatus*	small	366	21	2
Acanthuridae	*Acanthurus lineatus*	small	1400	23	2
Acanthuridae	*Acanthurus nigricauda*	small	385	34	1
Acanthuridae	*Acanthurus olivaceus*	small	310	31	1
Acanthuridae	*Acanthurus triostegus*	small	2963	19	1
Acanthuridae	*Acanthurus xanthopterus*	large	653	50	1
Acanthuridae	*Ctenochaetus striatus*	small	456	21	3
Acanthuridae	*Naso caesius*	large	131	46	4
Acanthuridae	*Naso hexacanthus*	large	154	60	1
Acanthuridae	*Naso lituratus*	small	2860	30	3
Acanthuridae	*Naso tonganus*	large	112	60	1
Acanthuridae	*Naso unicornis*	large	2964	59	1
Acanthuridae	*Naso vlamingii*	large	217	50	1
Lethrinidae	*Gnathodentex aureolineatus*	small	633	32	1
Lethrinidae	*Gymnocranius microdon*	small	68	41	5
Lethrinidae	*Lethrinus amboinensis*	medium	45	50	1
Lethrinidae	*Lethrinus atkinsoni*	small	550	42	1
Lethrinidae	*Lethrinus erythracanthus*	large	83	75	1
Lethrinidae	*Lethrinus harak*	small	2686	40	1
Lethrinidae	*Lethrinus obsoletus*	small	2004	40	1
Lethrinidae	*Lethrinus olivaceus*	large	623	79	1
Lethrinidae	*Lethrinus rubrioperculatus*	medium	3929	47	2
Lethrinidae	*Lethrinus xanthochilus*	large	932	70	1
Lethrinidae	*Monotaxis grandoculis*	medium	431	51	1
Lutjanidae	*Aphareus furca*	small	357	40	1
Lutjanidae	*Aprion virescens*	large	599	114	1
Lutjanidae	*Lutjanus argentimaculatus*	medium	208	73	1
Lutjanidae	*Lutjanus bohar*	large	324	93	1
Lutjanidae	*Lutjanus fulvus*	small	1444	35	1
Lutjanidae	*Lutjanus gibbus*	medium	352	50	1
Lutjanidae	*Lutjanus kasmira*	small	1562	34	1
Lutjanidae	*Lutjanus monostigma*	medium	644	52	1
Lutjanidae	*Macolor niger*	medium	68	59	1
Scaridae	*Calotomus carolinus*	small	166	33	1
Scaridae	*Cetoscarus ocellatus*	large	79	51	1
Scaridae	*Chlorurus frontalis*	large	404	49	1
Scaridae	*Chlorurus microrhinos*	large	564	57	1
Scaridae	*Chlorurus spilurus*	small	1511	37	1
Scaridae	*Hipposcarus longiceps*	large	794	62	1
Scaridae	*Scarus altipinnis*	large	582	51	1
Scaridae	*Scarus festivus*	small	78	43	1
Scaridae	*Scarus forsteni*	small	215	39	2
Scaridae	*Scarus ghobban*	large	161	56	1
Scaridae	*Scarus psittacus*	small	844	34	2
Scaridae	*Scarus rubroviolaceus*	large	272	54	1
Scaridae	*Scarus schlegeli*	small	1272	40	1
Serranidae	*Cephalopholis argus*	medium	237	46	2
Serranidae	*Cephalopholis sonnerati*	medium	404	50	5
Serranidae	*Cephalopholis urodeta*	small	742	28	4
Serranidae	*Epinephelus fasciatus*	small	3176	31	2
Serranidae	*Epinephelus hexagonatus*	small	380	27	1
Serranidae	*Epinephelus howlandi*	medium	58	47	1

Serranidae	*Epinephelus merra*	small	2179	32	1
Serranidae	*Epinephelus polyphekadion*	medium	117	62	1
Serranidae	*Epinephelus tauvina*	medium	48	52	1
Serranidae	*Plectropomus laevis*	large	70	84	1
Serranidae	*Variola albimarginata*	medium	314	60	1
Serranidae	*Variola louti*	medium	893	66	1

Supporting Information 1. Fish body-size groupings for dominant families. Jenks breaks were used to determine cut points within each family based upon maximum lengths (L_{max}). Two or three Jenks breaks categories were used depending upon the range of body-sizes within each family. Higher trophic level families such as snappers and groupers had a greater range of sizes and were broken into three classes, while herbivore/detritivore families had a limited range of size and were broken into two classes. Body-size ratios within each family were calculated by taking the biomass of medium-and-large species and dividing by the biomass of small species. L_{max} were preferentially derived from the present dataset (1), however, when values were not in agreement with similar local studies potentially due to reporting errors in the creel dataset (*see methods*), values were gathered from (2) Guam fisheries dependent data (Kamikawa et al. 2015), (3) recent CNMI fisheries dependent data (Matthews et al. 2017), (4) past CNMI data (Graham 1994, Houk et al. 2012), and (5) unpublished Micronesia data from author JCB, respectively.

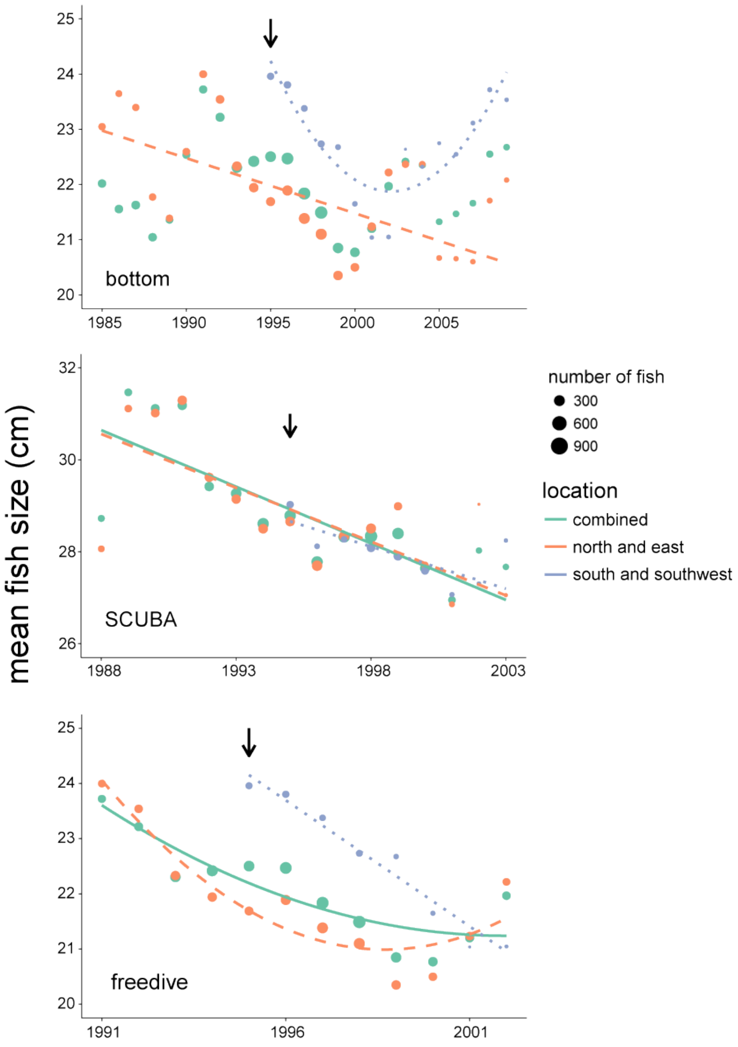

Supporting Information 2. Preliminary analyses of mean fish sizes versus fishing location during the study period. The construction of a new boat marina on the southwest side of the island in 1995 represented a fishery expansion (black arrows) that appeared to benefit both the bottom and freedive fishery. However, the SCUBA fishery did not show similar increases in fish size in the south and southwest with the expansion. Best-fit regression lines are shown for all significant relationships. Only polynomial and linear models fits existed (*see methods*).

References:

Graham, T. 1994. Biological analysis of the nearshore reef fish fishery of Saipan and Tinian. CNMI Division of Fish and Wildlife Technical Report **94**-02.

Houk, P., K. Rhodes, J. Cuetos-Bueno, S. Lindfield, V. Fread, and J. McIlwain. 2012. Commercial coral-reef fisheries across Micronesia: A need for improving management. Coral Reefs **31**:13-26.

Kamikawa, K., E. Cruz, T. Essington, J. Hospital, J. Brodziak, and T. Branch. 2015. Length–weight relationships for 85 fish species from Guam. Journal of Applied Ichthyology **31**:1171-1174.

Matthews, T., J. Gourley, A. Flores, M. Ramon, and M. Trianni. 2017. Length-weight relationships for 83 reef and bottomfish species from the Commonwealth of the Northern Mariana Islands (CNMI). Pacific Islands Fishery Science Center, National Marine Fisheries Service, NOAA, Honolulu, HI.:12.
